# Towards Safer Water: A Low‐Cost Disposable Electrochemical Sensor for Bisphenol A Using La_2_Sn_2_O_7_ Nanostructures

**DOI:** 10.1002/gch2.202500579

**Published:** 2026-01-14

**Authors:** Ragu Sasikumar, Balasubramanian Akila, Shen‐Ming Chen, Jongwon Kim, Byungki Kim

**Affiliations:** ^1^ School of Mechatronics Engineering Advanced Technology Research Center Korea University of Technology and Education Cheonan Chungnam Republic of Korea; ^2^ Centre of Molecular Medicine and Diagnostics Saveetha Dental College and Hospitals Saveetha Institute of Medical and Technical Sciences Saveetha University Chennai India; ^3^ Department of Chemical Engineering and Biotechnology National Taipei University of Technology Taipei Taiwan; ^4^ Department of Mechanical Design Engineering Korea University of Technology and Education Cheonan Chungnam Republic of Korea; ^5^ Future Convergence Engineering Korea University of Technology and Education Cheonan Chungnam Republic of Korea

**Keywords:** bisphenol A, electrocatalytic sensor, environmental monitoring, lanthanaum stannate, water sample analysis

## Abstract

Bisphenol A (BPA), a widely used industrial chemical, persists in aquatic environments and poses serious endocrine‐disrupting risks to ecosystems and human health. This study presents a highly sensitive, selective electrochemical sensor using lanthanum stannate (La_2_Sn_2_O_7_), a rare‐earth stannate, engineered for efficient BPA detection in real water matrices. The La_2_Sn_2_O_7_ nanostructure was synthesized and employed as an electrocatalytic modifier, offering unique physicochemical properties that facilitate accelerated electron transfer kinetics and abundant electroactive surface sites. Systematic electrochemical characterizations confirmed the material's superior catalytic performance, attributable to its synergistic structural and electronic attributes. Under optimized pH and operating conditions, the La_2_Sn_2_O_7_‐modified electrode demonstrated exceptional analytical capability, exhibiting an ultra‐low detection limit of 1.4 nM and a broad linear dynamic range spanning 0.001‒425.8 µm. These findings indicate remarkable sensitivity and reliability in quantifying trace BPA levels. Moreover, the sensor demonstrated excellent analytical recovery and reproducibility in diverse real‐water samples, including lake, river, tap, and plastic‐bottled water, underscoring its robustness and practical applicability in complex environmental matrices. La_2_Sn_2_O_7_ shows strong promise as an electrocatalyst, enabling real‐time BPA detection for enhanced environmental monitoring and public health protection in the field.

## Graphical abstract

A highly sensitive electrochemical sensor featuring lanthanum stannate nanomaterial is introduced for the rapid detection of bisphenol A (BPA) in water. The sensor delivers fast response, excellent selectivity, and ultra‐low detection limits, enabling reliable monitoring of BPA in real environmental samples. This technology offers a promising tool for safeguarding water quality and public health.

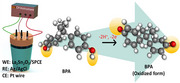

## 1 Introduction

**SCHEME 1:**
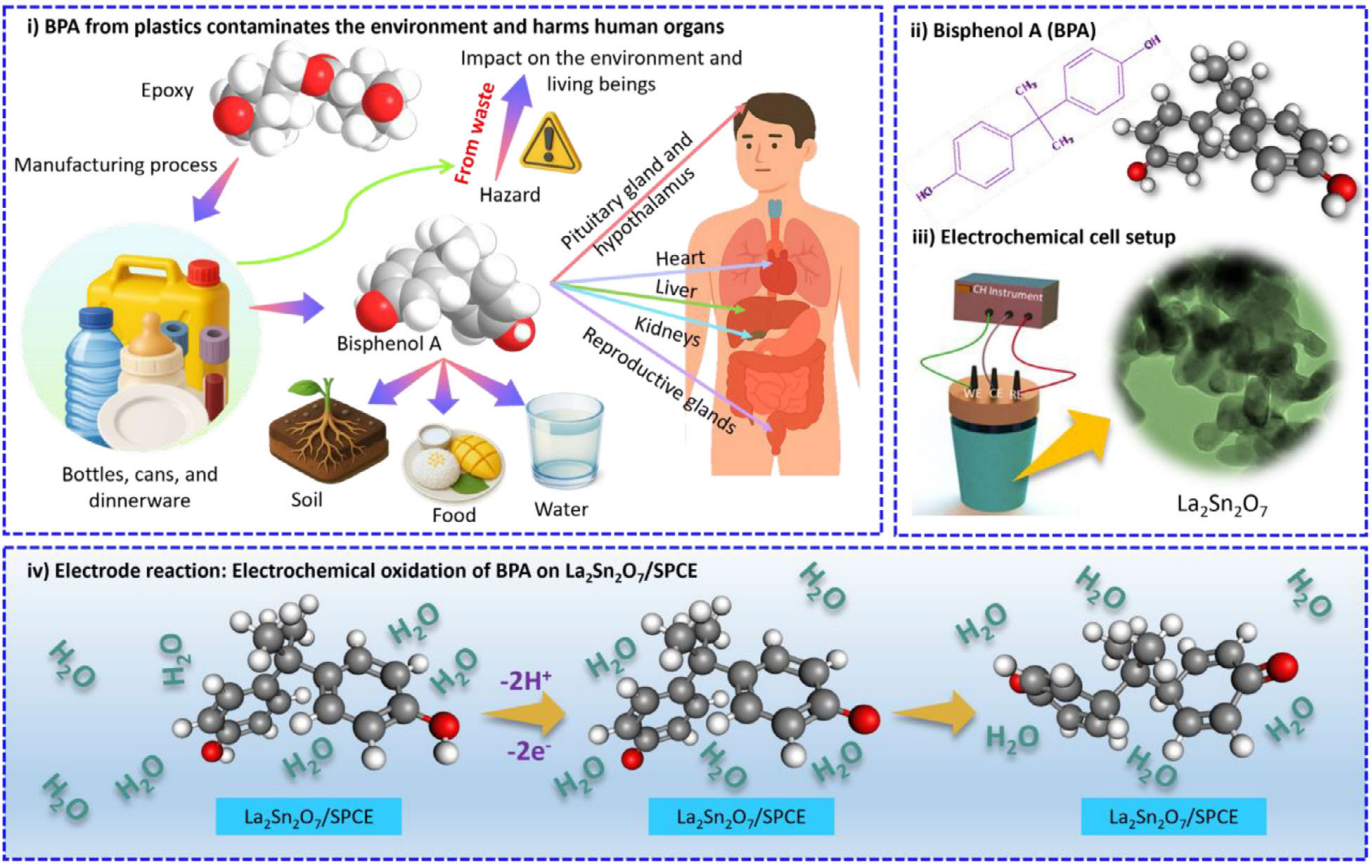
Schematic illustration of: (i) the pathway of BPA contamination from plastics into the environment and its harmful effects on human organs, (ii) the molecular structure of BPA, (iii) the electrochemical three‐electrode setup of La_2_Sn_2_O_7_, and (iv) the electrochemical oxidation mechanism of BPA on the fabricated La_2_Sn_2_O_7_/SPCE.

Bisphenol A (BPA), an organic compound belonging to the family of diphenylmethane derivatives, is a widely used industrial chemical primarily employed in the synthesis of polycarbonate plastics, epoxy resins, and certain types of flame retardants [1]. These materials are extensively utilized in the production of everyday consumer items such as beverage bottles, food storage containers, medical devices, and inner coatings of metal cans [2]. However, due to weak covalent linkages between BPA monomers within these polymers, leaching and migration readily occur during thermal stress, hydrolysis, or photodegradation [3]. Consequently, BPA is continuously released into various environmental compartments, particularly into surface water, ground water, sediments, and soil through industrial effluents, landfill leachates, and wastewater treatment discharges (Scheme 1i) [4]. Once introduced into the environment, BPA (Scheme 1ii) exhibits limited biodegradability and high environmental persistence, leading to chronic exposure and bioaccumulation through the trophic chain. As depicted in Scheme 1, the entire lifecycle of BPA, from its industrial production and product disposal to its infiltration into environmental media and subsequent human exposure‐illustrates the complexity of its contamination pathways. BPA contamination in soil and aquatic ecosystems contributes directly to the contamination of agricultural produce and drinking water sources. Upon entering the human body through ingestion, dermal adsorption, or inhalation, BPA acts as a potent endocrine‐disrupting compound (EDC) capable of binding to estrogen receptors (ERα, ERβ), androgen receptors, and thyroid hormone receptors [5]. This molecular mimicry results in significant endocrine dysregulation, which has been associated with infertility, obesity, cardiovascular dysfunctions, metabolic disorders, neurotoxicity, and carcinogenesis. The figure further outlines BPA's systematic toxicological effects, affecting the pituitary gland, hypothalamus, liver, heart, kidneys, and reproductive glands, highlighting the extensive physiological burden imposed by prolonged exposure [6, 7, 8, 9]. To mitigate these effects, the scheme also illustrates current detection methods (chromatographic [10, 11], immunoassay [12, 13], and electrochemical sensors [14, 15]) and remediation strategies (biodegradation [16], membrane filtration [17], oxidative degradation [18], and adsorption [19], under investigation. Despite ongoing efforts, the trace‐level quantification and continuous monitoring of BPA in environmental water and soil remain a formidable challenge due to matrix interferences, low pollutant concentrations, and the requirement for portable, cost‐effective analytical devices capable of in‐field operation [20, 21, 22, 23, 24, 25, 26, 27, 28].

The performance of electrochemical sensors for BPA detection (Scheme 1iii,iv) strongly depends on the physicochemical and electronic properties of the electrode materials. Recently, lanthanum stannate (La_2_Sn_2_O_7_), a rare‐earth stannate with a cubic pyrochlore‐type structure, has gained attention as a promising sensing material owing to its exceptional electrocatalytic activity, chemical stability, and electronic conductivity [29]. Structurally, La_2_Sn_2_O_7_ consists of a 3D network of corner‐sharing SnO_6_ octahedra stabilized by La^3+^ ions. This configuration ensures high structural integrity and excellent oxygen ion mobility, facilitating rapid electron and ion transport across the lattice. The incorporation of La^3+^ introduces lattice strain and oxygen vacancies, which act as active catalytic centers for surface redox reactions, while the Sn^4+^/Sn^2+^ redox couple enhances charge‐transfer kinetics and overall electrocatalytic performance [30, 31]. At the nanoscale, La_2_Sn_2_O_7_ offers a large specific surface area and a high density of active sites that promote efficient adsorption and interaction with BPA molecules via *π*–*π* stacking and hydrogen bonding. These features accelerate BPA oxidation at lower overpotentials, yielding distinct current responses suitable for quantitative detection. Compared with conventional metal oxides such as TiO_2_, ZnO, and SnO_2_, La_2_Sn_2_O_7_ demonstrates superior durability, thermal stability, and resistance to electrode fouling‐crucial for long‐term analytical reliability. Additionally, the strong Lewis acidity of La^3+^ ions enhances electron‐transfer efficiency and catalytic sensitivity. Hence, integrating La_2_Sn_2_O_7_ nanostructures into electrochemical platforms enables high signal amplification, lower detection limits, and improved selectivity toward BPA even in complex matrices such as wastewater or soil extracts.

Conventional analytical methods‐such as high‐performance liquid chromatography (HPLC), fluorescence spectrophotometry, and gas chromatography‐mass spectrometry (GC‐MS) provide high precision but require costly instrumentation, extensive sample pretreatment, and skilled operation, limiting their use for in‐field analysis. In contrast, electrochemical techniques offer rapid, cost‐effective, and miniaturizable platforms capable of direct electron‐transfer detection with minimal sample processing [23, 24, 25, 26, 27, 28]. Electrochemical sensors detect BPA through its electro‐oxidation into phenoxy radicals and quinone‐type intermediates, generating a measurable current proportional to analyte concentration. Incorporation of La_2_Sn_2_O_7_ nanomaterials enhances the electrode's catalytic activity, electron mobility, and signal‐to‐noise ratio, achieving detection limits in the nanomolar range [29, 30, 31, 32]. The advent of portable electrochemical strip sensors, employing screen‐printed electrodes and compact potentiostats, has further advanced environmental monitoring by enabling on‐site, real‐time, and user‐friendly analysis. These sensors are low‐cost, energy‐efficient, and compatible with microfluidic or wireless systems, making them ideal for field deployment [33, 34, 35, 36].

**SCHEME 2:**
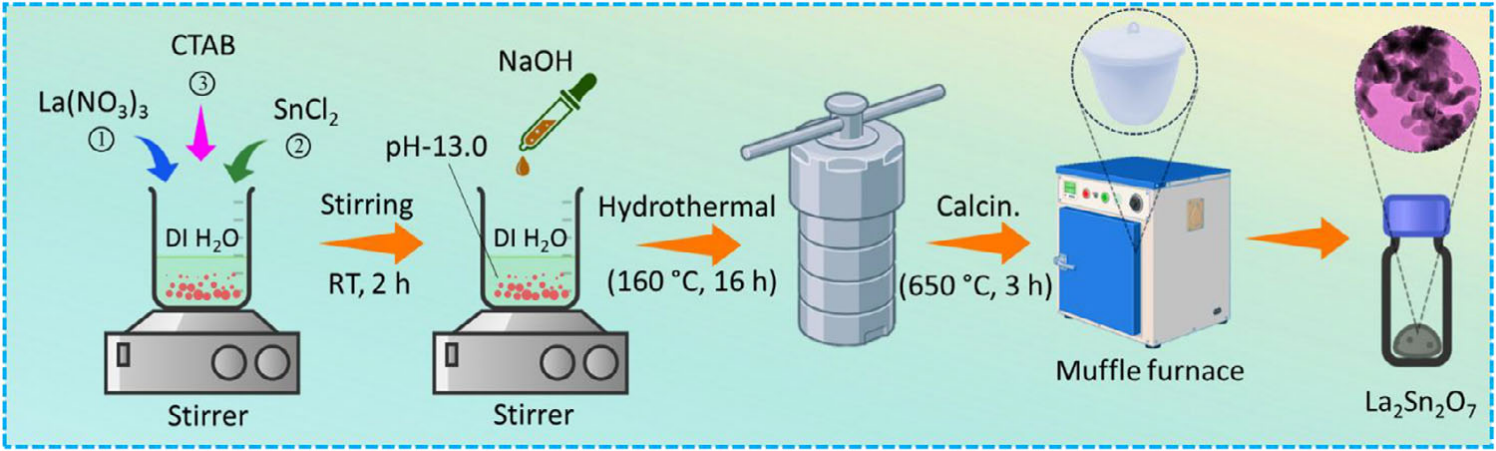
Schematic illustration of the synthesis of La_2_Sn_2_O_7_ nanoparticles via the hydrothermal route.

In this study, several analytical tests revealed the successful synthesis of a lanthanum stannate (La_2_Sn_2_O_7_) utilizing a hydrothermal procedure followed by simple stirring. In addition to outlining the synthesis, this study draws attention to certain unique features of the La_2_Sn_2_O_7_: (i) La_2_Sn_2_O_7_ possesses a stable cubic pyrochlore structure that ensures high structural integrity and electrochemical stability during BPA detection, (ii) its 3D network of SnO_6_ octahedra provides efficient electron and ion transport, enhancing charge‐transfer kinetics at the electrode interface, (iii) abundant oxygen vacancies and surface defects acts as active sites for BPA adsorption and redox catalysis, improving sensitivity, (iv) the nanostructured morphology offers a large surface are that promotes strong *π*–*π* and hydrogen‐bonding interactions with BPA molecules, (v) the coexistence of Sn^4+^/Sn^2+^ redox couples and lewis acidic La^3+^ centers accelerates electron transfer and amplifies electrochemical signals, and (vi) La_2_Sn_2_O_7_ exhibits excellent anti‐fouling and chemical durability, ensuring long‐term reproducibility and stability in complex environmental sample. In order to facilitate quick diagnosis in environmental and drinking water samples, the synthesized materials were attached to a disposable strip of a screen‐printed carbon electrode (SPCE) and used for electrochemical detection of BPA. The modified electrode showed significant electrochemical reaction activity toward BPA, with an impressive linear range (WLR) of 0.001–425.8 µm and a limit of detection (LOD) of 0.0014 µm. In addition to this, the improved sensor demonstrated remarkable selectivity, repeatability, and operational stability. Table S1 describes BPA's chemical structure and physical properties, and Scheme 2 shows the composite's fabrication route.

## 2 Experimental Section

### 2.1 Chemicals and Reagents

Lanthanum(III) nitrate hydrate (La(NO_3_)_3_·xH_2_O; CAS: 100587‐94‐8; 99.9%), tin chloride (SnCl_2_, CAS: 7772‐99‐8; 98%), cetyltrimethylammonium bromide (C_19_H_42_BrN; CTAB; CAS: 57‐09‐0), furaltadone (C_13_H_16_N_4_O_6_; CAS: 139‐91‐3), theobromine (C_7_H_8_N_4_O_2_; CAS: 83‐67‐0), dopamine hydrochloride ((HO)_2_C_6_H_3_CH_2_CH_2_NH_2_. HCl; CAS: 62‐31‐7), D‐(+)‐glucose (C_6_H_12_O_6_; CAS: 50‐99‐7; ≥99.5%), mefenamic acid (C_15_H_15_NO_2_; CAS: 61‐68‐7), carbendazim (C_9_H_9_N_3_O_2_; CAS: 10605‐21‐7; 97%), metol (C_7_H_9_NO. 1/2H_2_SO_4_; CAS: 55‐55‐0; ≥98%), lead(II) nitrate (Pb(NO_3_)_2_; CAS: 10099‐74‐8; ≥99.0%), mercury(II) nitrate monohydrate (Hg(NO_3_)_2_. H_2_O; CAS: 7783‐34‐8; ≥98.5%), L‐tryptophan (C_11_H_12_N_2_O_2_; CAS: 73‐22‐3; 99.0‐101.0%), sodium hydroxide (NaOH; NaOH; CAS: 1310‐73‐2), ethanol (EtOH; CH_3_CH_2_OH; CAS: 64‐17‐5) were used and purchased from Sigma–Aldrich Inc., Taiwan. A 0.1 m phosphate buffer solution (PBS) was prepared at different pH levels (3.0, 5.0, 7.0, 9.0, and 11.0) using NaH_2_PO_4_ and Na_2_HPO_4_. The pH of each solution was adjusted as needed with NaOH or HCl. DI water (Milli‐Q, 18.2 MΩ) was used for all experiments.

### 2.2 Synthesis of La_2_Sn_2_O_7_

The synthesis employed La(NO_3_)_3_, SnCl_2_, and CTAB as precursor materials [31]. Initially, stoichiometric amounts of lanthanum nitrate and tin chloride were dissolved in 35 mL of DI water, followed by the addition of CTAB. The CTAB acted as a structure‐directing agent to facilitate the formation of anisotropic metallic nanostructures. NaOH was used to change the substance's pH to 13 media. After a 2 h vigorous stirring period at room temperature (RT), the resulting mixture was shifted to a stainless‐steel autoclave lined with 200 mL of Teflon. The autoclave was kept at a temperature of 160°C for 16 h, and then it was cooled down to RT. The resulting suspension was centrifugally cleaned multiple times with ethanol and deionized water to eliminate any remaining contaminants. After being dried at 80°C for 24 h, the product was calcinated at 650°C for 3 h.

### 2.3 Fabrication of La_2_Sn_2_O_7_ Nanoparticles on SPCE

**SCHEME 3:**
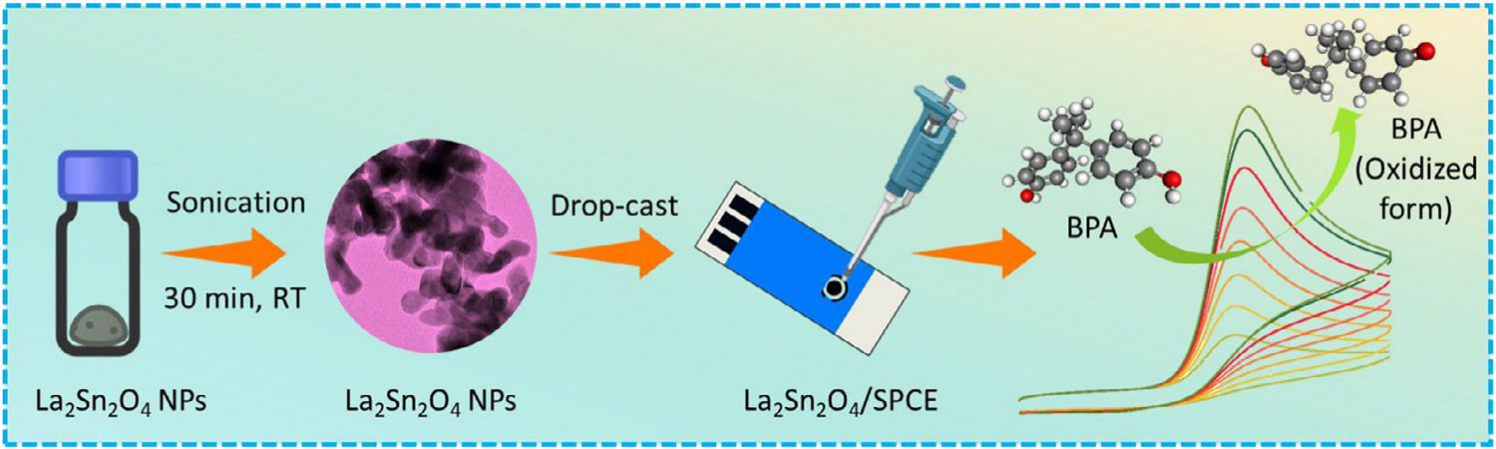
Schematic illustration of the fabrication of La_2_Sn_2_O_7_/SPCE towards BPA detection.

To modify the bare SPCE, 15.0 mg of the synthesized La_2_Sn_2_O_7_ nanoparticles was dispersed in 2.0 mL of DI water and subjected to ultrasonication for 30 min to obtain a homogeneous suspension. Subsequently, 8 µL of this suspension was drop‐cast onto the pre‐cleaned working surface of the SPCE and allowed to dry to RT. Voltammetric measurements were then conducted using the modified electrode (Scheme 3). For comparison, the same procedure was followed for the bare SPCE. The remaining portion of the nanoparticle suspension was stored at RT for further use.

### 2.4 Material Characterization and Electrochemical Testing

The surface morphology was examined using high‐resolution transmission electron microscopy (HR‐TEM, JEM‐2100 Plus, JEOL) equipped with an energy‐dispersive X‐ray spectroscopy (EDX) system. The crystalline structures were characterized by powder X‐ray diffraction (PXRD; Malvern PANalytical, United Kingdom) with Cu Kα radiation (λ = 1.54 Å). Fourier transform infrared (FT‐IR) spectra were recorded at room temperature on a Shimadzu IR Tracer 100 spectrophotometer using KBr pellets, with a spectral resolution of 2 cm^−1^. The composition and elemental electronic states of the La_2_Sn_2_O_7_ composite were analyzed by X‐ray photoelectron spectroscopy (XPS; PHI VersaProbe III Scanning XPS Microprobe, Physical Electronics, USA). Electrochemical oxidation of BPA was carried out under N_2_‐saturated conditions using a three‐electrode system, which included a modified screen‐printed carbon electrode (SPCE) as the working electrode (WE), a saturated Ag/AgCl as the reference electrode (RE), and a platinum wire as the counter electrode (CE). The electrochemical behavior was evaluated through cyclic voltammetry (CV) and amperometry (i‐t curve) using a CHI electrochemical workstation (CHI 1211c). Additionally, electrochemical impedance spectroscopy (EIS) measurements were performed with an AUTOLAB PGSTAT101.

## 3 Results and Discussion

### 3.1 Morphological Structure

**FIGURE 1:**
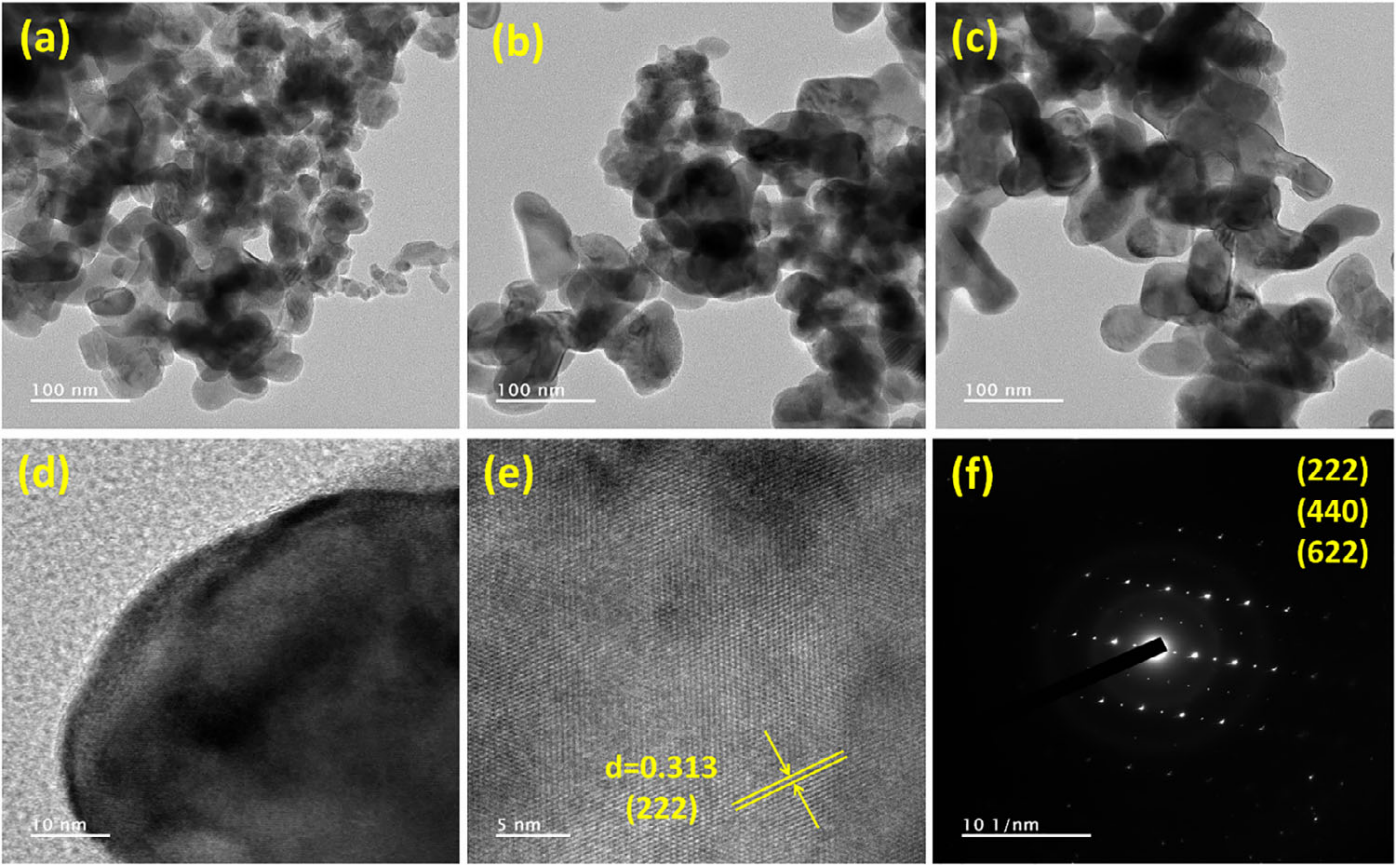
(a–c) HR‐TEM images of La_2_Sn_2_O_7_ nanoparticles, (d) crystalline structure, (e) corresponding lattice fringes, and (f) SAED pattern of La_2_Sn_2_O_7_ nanoparticles.

The microstructural and crystallographic features of the synthesized La_2_Sn_2_O_7_ nanoparticles were thoroughly examined by HR‐TEM, and the corresponding images are displayed in Figure 1a–f. The low‐magnification HR‐TEM images shown in Figure 1a–c reveal that the product consists of aggregated nanoparticles with irregular yet predominantly spherical morphologies. The average particle size is estimated to lie within the range of 20–50 nm, as inferred from direct measurements of several individual particles. The agglomeration behavior observed in these micrographs can be attributed to the high surface energy and strong interparticle van der Waals forces that typically develop during the calcination and post‐synthesis drying processes. Similar agglomeration is often reported in rare‐earth‐based pyrochlore oxides due to their high reactivity and surface energy. A more magnified HR‐TEM image presented in Figure 1d focuses on a single La_2_Sn_2_O_7_ nanoparticle, exhibiting a relatively uniform contrast and well‐defined grain boundary, indicating the presence of a single crystalline domain rather than an amorphous or polycrystalline aggregate. The well‐developed crystalline edges confirm that the particles maintain a thermodynamically stable morphology after calcination. The internal structure shows clear periodic fringes, implying a high degree of crystallinity within the nanoparticle matrix. The HR‐TEM image shown in Figure 1e provides detailed insight into the crystal structure of the synthesized La_2_Sn_2_O_7_ nanoparticles. Well‐defined and periodic lattice fringes are clearly visible, confirming the formation of a highly ordered crystalline lattice. The measured interplanar spacing (d = 0.313 nm) corresponds to the (222) plane of cubic La_2_Sn_2_O_7_, consistent with the XRD results and standard JCPDS data. The uniform and continuous lattice fringes indicate the absence of structural defects or amorphous regions, reflecting the excellent crystallinity of the sample. The SAED pattern shown in Figure 1f exhibits sharp, concentric diffraction rings indexed to the (222), (440), and (622) planes, characteristic of the cubic pyrochlore phase. The distinct and well‐defined rings confirm the nanocrystalline and single‐phase nature of the synthesized material. Overall, the HR‐TEM and SAED analyses demonstrate that the prepared La_2_Sn_2_O_7_ nanoparticles possess a highly crystalline pyrochlore structure (space group Fd‐3m) with uniform morphology and well‐ordered lattice planes. These structural features confirm the efficiency of the synthesis route and suggest potential suitability for catalytic and energy‐related applications where high crystallinity and surface uniformity are advantageous.

### 3.2 Structural Studies

**FIGURE 2:**
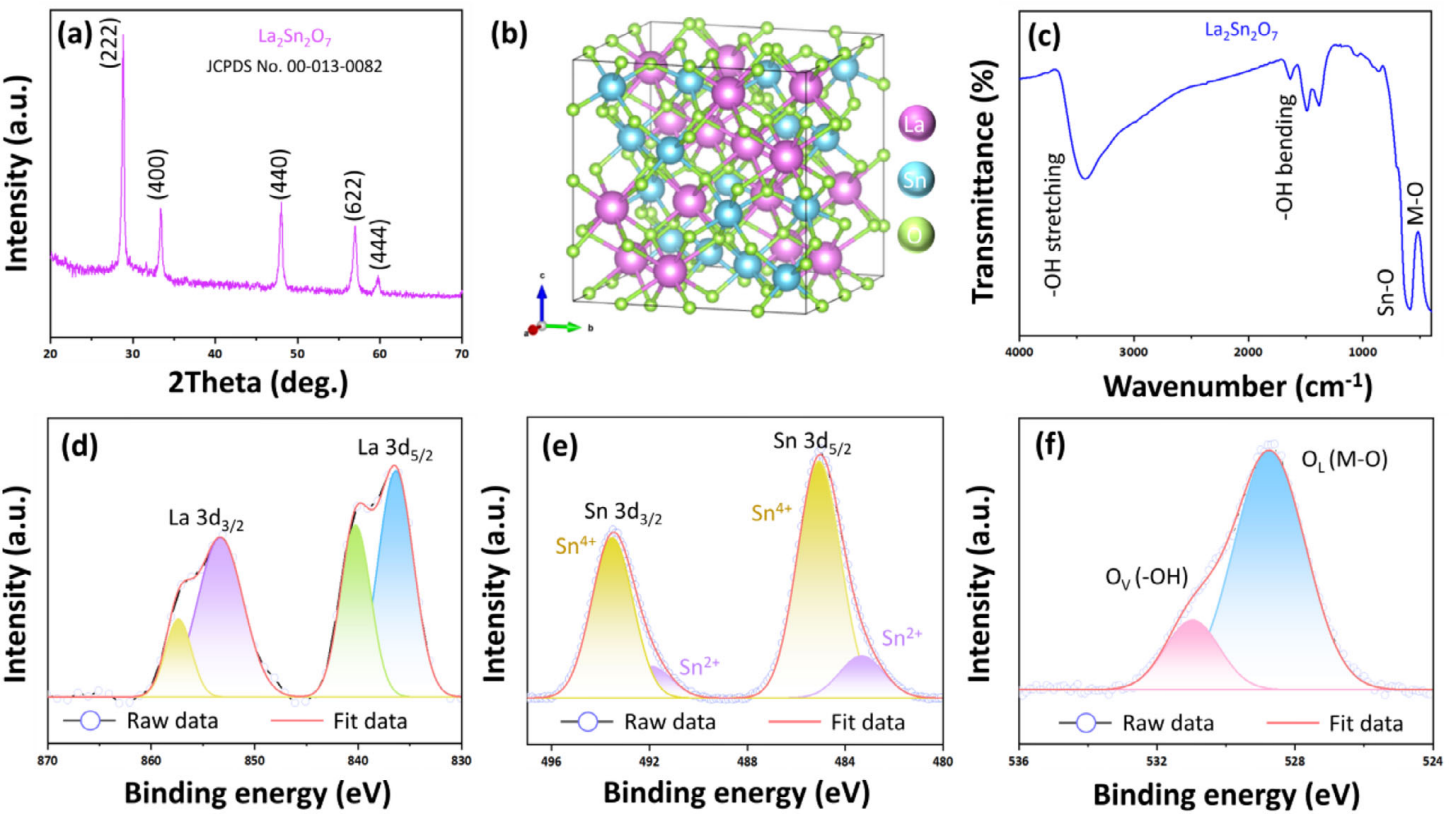
(a) XRD patterns, (b) crystal structure, (c) FT‐IR spectrum of La_2_Sn_2_O_7_, and XPS high‐resolution spectra of (d) La 3d, (e) Sn 3d, and (f) O 1s.

The phase composition and crystallinity of the synthesized La_2_Sn_2_O_7_ were examined using XRD, as shown in Figure 2a. All diffraction peaks are sharp and well‐defined, corresponding to the characteristic reflections of the cubic pyrochlore structure (space group *Fd‐3m*) with lattice symmetry matching the standard JCPDS card No. #00‐013‐0082. The prominent peaks located and indexed to 28.78° (222), 33.29° (400), 48.06° (440), 56.98° (622), and 59.8° (444) can be planes [29]. The absence of any additional reflections confirms the formation of a single‐phase La_2_Sn_2_O_7_ pyrochlore structure without detectable secondary phases such as La_2_O_3_ or SnO_2_. The narrow peak widths indicate high crystallinity and well‐developed long‐range ordering. The average crystallite size can be estimated from the Scherrer equation, typically yielding values within the 20–40 nm range for similar pyrochlore stannates. The structural stability of La_2_Sn_2_O_7_ arises from the ordered arrangement of La^3+^ at the A‐site and Sn^4+^ at the B‐site within a 3D network of corner‐shared SnO_6_ octahedra, producing intrinsic oxygen vacancies that can influence electrical and catalytic properties. Recent investigations have reported comparable XRD features for La_2_Sn_2_O_7_ prepared via the hydrothermal method, confirming the robustness of this pyrochlore framework and its potential as a stable mixed‐oxide material. The crystal structure of the La_2_Sn_2_O_7_ nanoparticle is visualized using VESTA software, as shown in Figure 2b.

The FT‐IR spectrum of La_2_Sn_2_O_7_ (Figure 2c) provides insight into the surface functional groups and metal‐oxygen bonding environment. The broad absorption band centered near ∼3430 cm^−1^, together with a weaker band ∼1645 cm^−1^, corresponds to the stretching and bending vibrations of O─H groups and H─O─H bending modes of adsorbed moisture, respectively. These features are commonly observed for oxide materials exposed to ambient conditions and are indicative of surface hydroxylation. In the low‐wavenumber region, strong absorption bands observed between ∼420, ∼594, and ∼855 cm^−1^ are attributed to metal‐oxygen (M‒O) vibrations associated with Sn─O stretching within SnO_6_ octahedra and La‒O lattice vibrations of the pyrochlore framework. The position and intensity of these M‒O modes confirm the integrity of the La_2_Sn_2_O_7_ lattice and are consistent with previously reported vibrational signatures of lanthanum stannate pyrochlores. The absence of carbonate‐related absorptions (∼1400–1500 cm^−1^) further indicates the effective removal of residual precursors during calcination. From a structural perspective, the distinct M‒O absorption features demonstrate that the La─O and Sn─O bonds remain well ordered within the crystal lattice, while the weak O‒H bands imply only minor surface hydroxylation. Such lattice integrity is crucial for maintaining the functional stability of La_2_Sn_2_O_7_, as oxygen‐related vibrations directly influence ionic conductivity, redox activity, and optical properties. Moreover, oxygen vacancies inherent to the pyrochlore lattice can broaden or slightly shift these M‒O vibrations, consistent with reports on defect‐mediated functionalities in rare‐earth stannates.

Figure S1 presents the spectra obtained from the XPS analysis of La_2_Sn_2_O_7_ nanoparticles, recorded over a binding energy range of 0‒1200 eV. The distinct peaks observed in the survey spectrum confirm the coexistence of La 3d (∼840 eV), Sn 3d (∼530 eV), and O 1s (∼490 eV) within the nanoparticle structure. The high‐resolution La 3d spectrum (Figure 2d) shows two characteristic peaks centered at ∼834.7 and ∼851.7 eV, corresponding to La 3d_5/2_ and La 3d_3/2_, respectively, indicating the presence of La^3+^ species. The Sn 3d spectrum (Figure 2e) exhibits major spin‐orbit doublets at ∼486.5 eV (Sn 3d_5/2_) and ∼495.1 eV (Sn 3d_3/2_), consistent with the oxidation state of Sn^4+^ in La_2_Sn_2_O_7_ nanoparticles. Similarly, the Sn 3d spectrum (Figure 2e) exhibits minor spin‐orbit doublets at ∼484.33 eV (Sn 3d_5/2_) and ∼494.52 eV (Sn 3d_3/2_), consistent with the oxidation state of Sn^2+^ in La_2_Sn_2_O_7_ nanoparticles. Furthermore, the deconvoluted O 1s spectrum (Figure 2f) reveals two primary components located near ∼530.6 and ∼532.4 eV, which can be attributed to lattice oxygen bond (O_L_, M‒O) and hydroxyl oxygen (Ov, ‒OH), respectively.

### 3.3 Electrochemical Performance of La_2_Sn_2_O_7_/SPCE

#### 3.3.1 EIS Studies of La_2_Sn_2_O_7_/SPCE in Redox Solution

**FIGURE 3:**
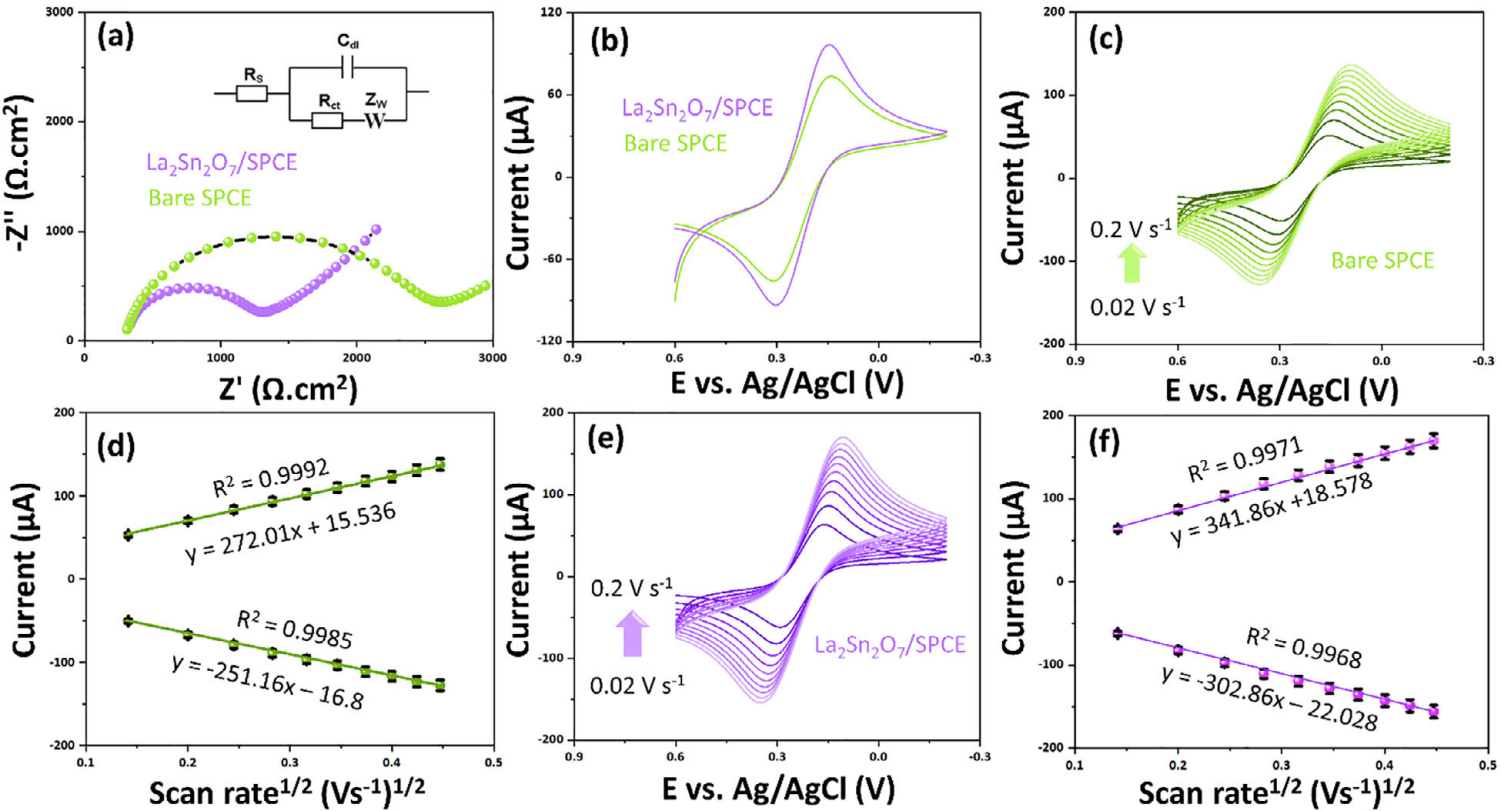
(a) EIS, (b) CVs were conducted in a 5.0 mM [Fe(CN)_6_]^3−/4−^ and 0.1 M KCl solution at various modified electrodes, (c–f) CVs were recorded at different scanning rates (0.02 to 0.2 V s^−1^), and the corresponding linear plots of redox peak currents vs. the square roots of the scanning rates (Error bars represent the standard deviation (SD) of scan rates).

EIS and CV were systematically employed to elucidate the charge transfer characteristics and interfacial electron transport behavior of the La_2_Sn_2_O_7_/SPCE in comparison with the bare SPCE (Figure 3a–f). EIS analysis (Figure 3a) exhibited semicircular Nyquist plots for both electrodes, confirming the dominance of charge transfer resistance (R_ct_) at the electrode‐electrolyte interface. The La_2_Sn_2_O_7_/SPCE electrode displayed a significantly smaller semicircle diameter than the bare SPCE, indicating a pronounced decrease in R_ct_ (La_2_Sn_2_O_7_/SPCE = 987.01 Ω·cm^2^ and bare SPCE = 2245.19 Ω·cm^2^) and hence an enhanced electron transfer rate. This reduction in R_ct_ can be attributed to the intrinsic electrical conductivity and defect‐rich nature of the La_2_Sn_2_O_7_ pyrochlore structure. The presence of abundant oxygen vacancies and mixed valence states (La^3+^/Sn^4+^ ↔ La^2+^/Sn^2+^) generates localized electronic states within the bandgap, facilitating rapid charge hopping and electron delocalization across the oxide lattice. Moreover, the 3D corner‐sharing SnO_6_ octahedral framework provides multiple conduction pathways for charge transport, leading to improved interfacial conductivity. These results in lower interfacial resistance and superior electrical conductivity, favoring efficient charge transfer during the redox process.

#### 3.3.2 CV Studies of La_2_Sn_2_O_7_/SPCE in Redox Solution

CV measurements (Figure 3b) in 5.0 mm [Fe(CN)_6_]^3−/4−^ solution further demonstrated the improved electrochemical response of La_2_Sn_2_O_7_/SPCE compared to the bare electrode (I_pa_ = 73.20 µA at E_pa_ = 0.139 V and I_pc_ = −75.92 µA at E_pa_ = 0.312 V). The La_2_Sn_2_O_7_/SPCE exhibited significantly higher redox peak currents of I_pa_ = 97.04 µA at E_pa_ = 0.149 V and I_pc_ = −94.32 µA at E_pa_ = 0.149 V and a smaller peak‐to‐peak separation (∆E_p_) as ∆E_p_ = 152 mV, confirming enhanced electron transfer kinetics and better reversibility of the redox process. The higher current response is attributed to the increased electrochemically active surface area (ECSA) and the high density of surface oxygen defects in La_2_Sn_2_O_7_, which facilitate stronger adsorption and faster charge exchange with the redox probe. Additionally, the presence of polar La─O and Sn─O bonds at the surface enhances electrostatic interactions with the negatively charged ferricyanide ions, improving electron coupling at the electrode‐solution interfaces. To probe the kinetic mechanism, CVs were recorded at different scanning rates (0.02 to 0.2 V s^−1^) for both electrodes (Figure 3c,e). In both instances, the anodic and cathodic peak currents increased in a manner that corresponds with the square root of the scanning rate (Figure 3d,f, and Equations (1), (2), (3), (4)). This suggests that the electrochemical reaction is controlled by diffusion rather than being limited by adsorption.

Bare SPCE:

(1) $$I_{pa}\;\left(\mu A\right)=272.01\nu \;{\left(\mathrm{mV}\;s^{-1}\right)}^{1/2}+15.536;\;\;R^{2}=0.9992$$

(2) $$I_{pa}\;\left(\mu A\right)=-251.16\nu \;{\left(\mathrm{mV}\;s^{-1}\right)}^{\frac{1}{2}}\;-\;16.8;\;\;R^{2}=0.9985$$

La_2_Sn_2_O_7_/SPCE:

(3) $$I_{pa}\;\left(\mu A\right)=341.86\nu \;{\left(\mathrm{mV}\;s^{-1}\right)}^{1/2}+18.578;\;\;R^{2}=0.9971$$

(4) $$I_{pa}\;\left(\mu A\right)=-302.86\nu \;{\left(\mathrm{mV}\;s^{-1}\right)}^{\frac{1}{2}}-\;22.028;\;\;R^{2}=0.9968$$

However, the La_2_Sn_2_O_7_/SPCE exhibited substantially higher slopes and correlation coefficients (R^2^ > 0.99) than the bare electrode, reflecting enhanced diffusion flux and rapid charge transport within the La_2_Sn_2_O_7_ matrix. The superior electrochemical performance of La_2_Sn_2_O_7_/SPCE is mechanistically attributed to its pyrochlore‐type crystal structure and oxygen‐deficient lattice, which provide multiple conduction channels and an increased density of active sites for electron transfer. The coexistence of La and Sn cations in different oxidation states enables a mixed electronic‐ionic conduction mechanism, while the high surface roughness and porosity increase the effective conduct area with the electrolyte. These synergistic effects result in faster interfacial charge transfer and higher current response. The combination of low R_ct_ (987.01 Ω·cm^2^), larger ECSA (A = 0.095 cm^2^), and stable diffusion‐controlled kinetics demonstrates that La_2_Sn_2_O_7_ is an efficient and stable electroactive material for BPA sensing applications.

### 3.4 Effect of Electrode Modification, Catalyst Loading, and pH Towards BPA

**FIGURE 4:**
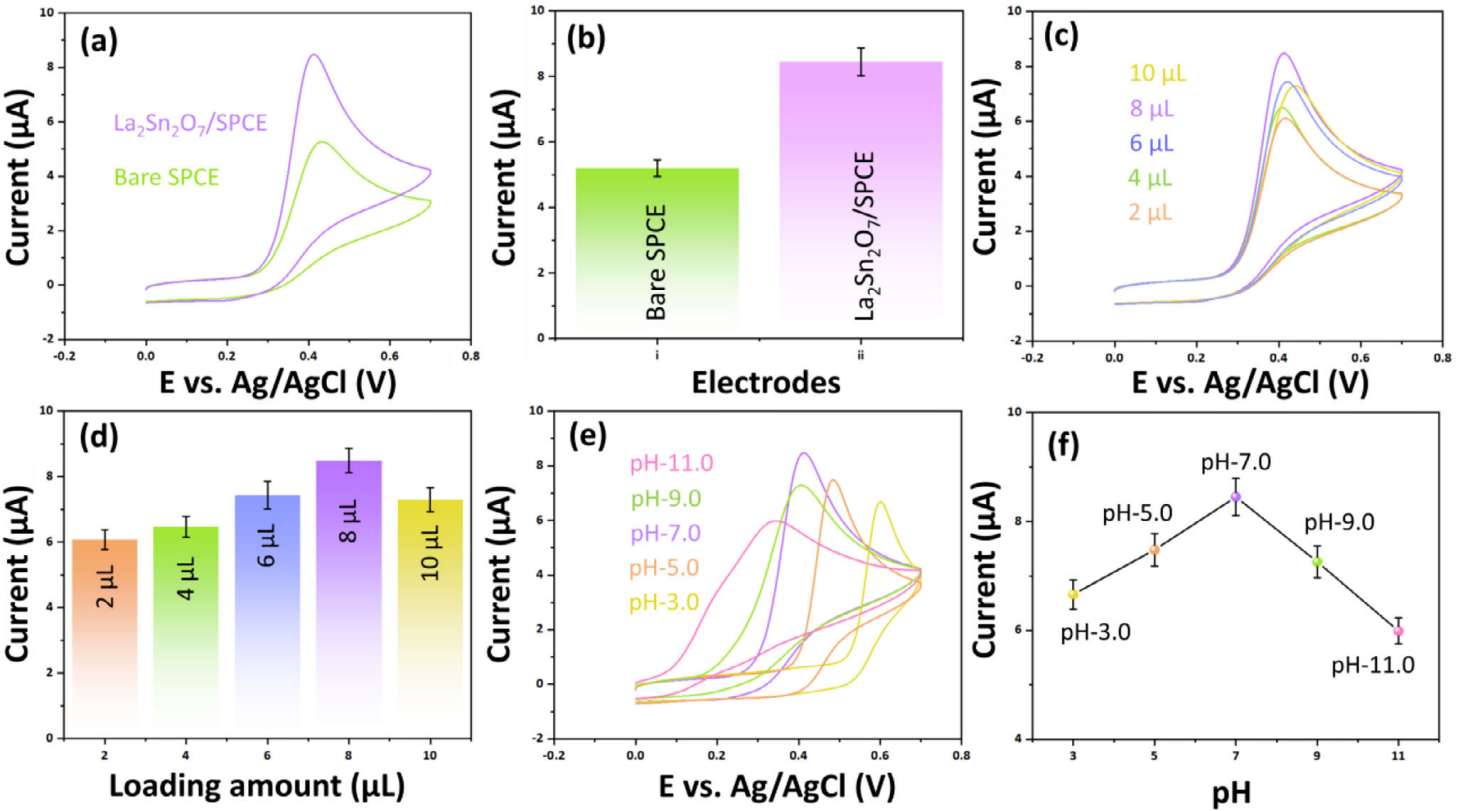
(a) CVs of different modified electrodes in the presence of BPA in 0.1 m PB (pH 7.0), (b) corresponding bar diagram showing the current response of different modified electrodes (Error bars represent the standard deviation (SD) of two independent measurements), (c) CVs illustrating the effect of La_2_Sn_2_O_7_ loading amount on electrode performance, studied by varying the deposition volume from 2 to 10 µL in the presence of BPA in 0.1 m PB (pH 7.0), (d) corresponding bar diagram showing the variation of current (µA) with loading amounts (µL) (Error bars represent the standard deviation (SD) of among 5 measurements), (e) effect of PB pH (3.0–11.0) on the CV response of the La_2_Sn_2_O_7_/SPCE, and (f) corresponding bar diagram showing current variation (µA) as a function of pH (Error bars represent the standard deviation (SD) of all pH).

The electrochemical behavior of the La_2_Sn_2_O_7_/SPCE was systematically evaluated toward the electrochemical detection of the BPA using CV in 0.1 m PB. A detailed investigation was carried out to understand the influence of electrode modification, catalyst loading, and electrolyte pH on the voltammetric response, thereby establishing the optimal operational parameters for BPA sensing. Figure 4a shows the comparative CV responses of the bare SPCE and La_2_Sn_2_O_7_/SPCE recorded in PBS containing BPA. The bare SPCE exhibited a weak redox signal with a low peak current of I_pa_ = 5.3 µA at 0.43 V, reflecting sluggish electron‐transfer kinetics and a limited number of active sites. In contrast, the La_2_Sn_2_O_7_/SPCE displayed a pronounced anodic peak and a significantly enhanced current response of I_pa_ = 8.49 µA at 0.4 V, indicating superior electrocatalytic activity toward BPA oxidation. As illustrated in Figure 4b, the current intensity of the modified electrode was markedly higher than that of the bare SPCE, confirming the beneficial role of La_2_Sn_2_O_7_ in promoting charge transfer. The enhanced electrochemical activity can be attributed to several synergistic factors. The La_2_Sn_2_O_7_ nanostructure provides a high specific surface area and abundant active sites for BPA adsorption, while its intrinsic electrical conductivity facilitates efficient charge transport between the analyte and the electrode. Moreover, the strong interaction between the phenolic hydroxyl groups of BPA and the surface oxygen species of La_2_Sn_2_O_7_ promotes rapid electron exchange. Consequently, the incorporation of La_2_Sn_2_O_7_ significantly improves both the sensitivity and reversibility of the BPA redox process, highlighting its suitability as an effective electrocatalyst for phenolic compound detection.

The influence of the La_2_Sn_2_O_7_ loading amount on the electrode performance was studied by varying the deposition volume from 2 to 10 µL (Figure 4c). The corresponding current variations are presented in Figure 4d. The oxidation current increased progressively with the loading volume up to 8 µL, beyond which a slight decrease was observed at higher loadings. This trend indicates that an optimal loading produces a uniform and conductive film, ensuring maximum exposure of catalytic sites and efficient electron transfer. However, excessive loading leads to particle agglomeration and a thicker film, which restricts ion diffusion and increases charge‐transfer resistance. Conversely, insufficient loading results in incomplete surface coverage, thereby reducing the number of accessible active sites. Thus, 8 µL was determined to be the optimal loading amount, providing a balance between effective surface coverage and charge transport efficiency. Under this condition, the electrode exhibited the highest current response and the most stable electrochemical behavior toward BPA oxidation.

The electrochemical response of BPA is strongly influenced by the pH of the supporting electrolyte due to the proton‐coupled nature of its redox process. The effect of pH on the CV response of La_2_Sn_2_O_7_/SPCE was examined over the range of 3.0 to 11.0 (Figure 4e). Both the anodic peak potential and current intensity varied systematically with pH. At acidic conditions (pH 3.0 to 5.0), the oxidation current was relatively low, which can be attributed to the high proton concentration impeding electron transfer and the reduced availability of deprotonated BPA species that participate in oxidation. As the pH increased to neutral (pH 7.0), the oxidation current reached its maximum value, indicating that the electrochemical oxidation of BPA proceeds most efficiently under near‐neutral conditions. Beyond pH 7.0, a gradual decline in current was observed (pH 9.0 to 11.0), likely due to the deprotonation of hydroxyl groups in BPA at higher pH levels, which diminishes its adsorption affinity on the electrode surface and hinders the catalytic process. The relationship between peak current and pH (Figure 4f) displays a bell‐shaped trend, with the highest current observed at pH 7.0. This pH‐dependent behavior confirms that the oxidation of BPA at La_2_Sn_2_O_7_/SPCE involves a proton‐coupled electron transfer mechanism and that proton availability plays a critical role in modulating the redox kinetics. Therefore, pH 7.0 was selected as the optimal condition for subsequent electrochemical studies.

### 3.5 Effect of Various BPA Concentrations and the Scanning Rates at La_2_Sn_2_O_7_/SPCE

**FIGURE 5:**
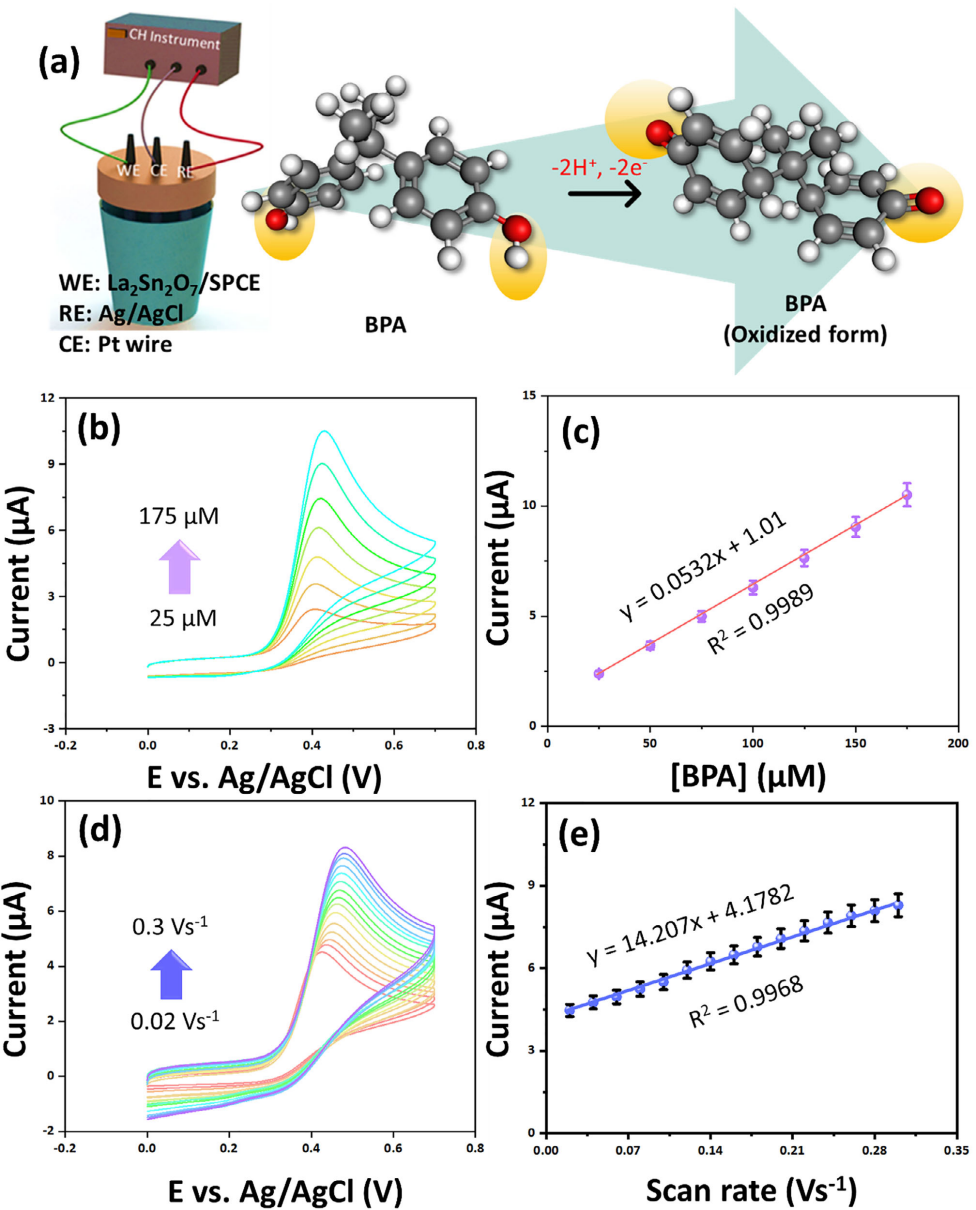
(a) Possible electro‐oxidation mechanism of BPA at La_2_Sn_2_O_7_/SPCE, (b) CVs obtained for varying BPA concentrations ranging from 25 to 175 µm in 0.1 m PB at a fixed scanning rate of 50 mV s^−1^ at La_2_Sn_2_O_7_/SPCE (Error bars represent the standard deviation (SD) of Concentration), (c) the corresponding linear relationship between the oxidation current (µA) and BPA concentration (µm), (d) CVs recorded at different scanning rates ranging from 0.02 to 0.3 V s^−1^ for a fixed BPA concentration of 150 µm, and (e) the corresponding linear relationship between the oxidation current (µA) and the scanning rates (Error bars represent the standard deviation (SD) of scanning rates).

In Figure 5a prospective BPA electro‐oxidation process at La_2_Sn_2_O_7_/SPCE. The electroactive phenolic groups of BPA undergo direct oxidation when subjected to anodic polarization, which ultimately leads to the creation of o‐quinone species being produced [14, 22, 23]. It is highly probable that there is a tautomeric form of oxidized BPA. This radical intermediate undergoes tautomeric conversions. Through a coupled two‐electron and two‐proton (2e^−^/2H^+^) transfer mechanism on the surface of La_2_Sn_2_O_7_/SPCE, quinoid products of BPA are generated in the final step of the process [37, 38, 39]. Figure 5b shows the CVs obtained for varying BPA concentrations ranging from 25 to 175 µm in 0.1 m PB at the fixed scanning rate of 50 mV s^−1^. With increasing BPA concentration, the anodic peak current gradually rises, indicating an enhanced electro‐oxidation process. This behavior can be attributed to the greater availability of electroactive BPA molecules near the electrode surface, facilitating electron transfer between BPA and the La_2_Sn_2_O_7_ nanostructure. The oxidation peak potential exhibits a slight positive shift with concentration, suggesting a quasi‐irreversible electron transfer process influenced by surface adsorption phenomena. The corresponding calibration plot in Figure 5b reveals a strong linear correlation between the oxidation current and BPA concentration, described by the regression with an excellent correlation coefficient Equation (5)

(5) $$I_{\mathrm{pa}}\;\left(\mu A\right)=0.0532\;\left[\mathrm{BPA}\;\left(\mu M\right)\right]+1.01;\;\;R^{2}=0.9989$$

The near‐unity R^2^ value demonstrates the high reproducibility and linear response of the modified electrode, confirming that BPA oxidation on La_2_Sn_2_O_7_/SPCE is primarily a concentration‐dependent process. The enhanced electrocatalytic activity can be attributed to the synergistic effect of La and Sn ions, which improve surface conductivity and provide abundant active sites for BPA adsorption and oxidation.

To gain insight into the reaction kinetics, CVs were recorded at different scanning rates ranging from 0.02 to 0.3 V s^−1^ for a fixed BPA concentration of 150 µm, as presented in Figure 5c. The anodic peak current increases progressively with increasing scanning rate, indicating accelerated charge transfer dynamics. A linear relationship between the peak current and the scanning rate is observed (Figure 5d), following the Equation (6)

(6) $$I_{\mathrm{pa}}\;\left(\mu A\right)=14.207\nu \;\left(\mathrm{mV}\;s^{-1}\right)+4.1782;\;R^{2}=0.9968$$

The linear dependence indicates that BPA electrochemical oxidation at the La_2_Sn_2_O_7_/SPCE electrode is diffusion‐controlled, instead of adsorption‐controlled.

### 3.6 Amperometric (i‐t), Selectivity, Reproducibility, and Operational Stability Performance of La_2_Sn_2_O_7_ Towards BPA

**FIGURE 6:**
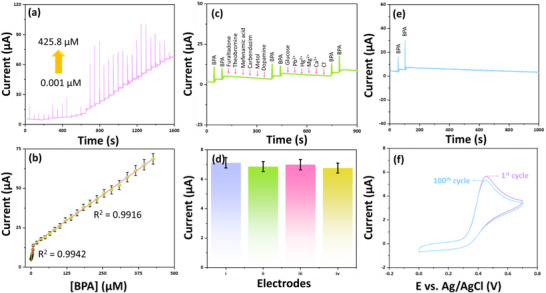
(a) The amperometric current‐time response of the La_2_Sn_2_O_7_/SPCE electrode exhibits a distinct stepwise increase with successive additions of BPA (0.001–425.8 µm) in 0.1 m PB (pH 7.0) at an applied potential of *E*_pa_ = 0.3 V, (b) the corresponding calibration plot shows the relationship between current response (µA) and BPA concentration (µm) (Error bars represent the standard deviation (SD) of among Concentration), (c) selectivity study of the La_2_Sn_2_O_7_/SPCE electrode in the presence of BPA with a 5‐fold excess of potential interfering compounds, (d) reproducibility (Error bars represent the standard deviation (SD) of 4 measurements (*n* = 4)), and (e,f) operational stability of the La_2_Sn_2_O_7_/SPCE electrode toward BPA.

The CV results confirm the excellent electrochemical performance of the La_2_Sn_2_O_7_ modified electrode toward the electrochemical oxidation of BPA. To further assess its sensing ability, the amperometry (i‐t) method was employed due to its high selectivity and sensitivity compared to other analytical methods. Under optimized electrochemical conditions, a rapid and amplified current response was observed upon successive additions of BPA, demonstrating the efficiency of the modified electrode. As shown in Figure 6a, the amperometric current‐time response of the La_2_Sn_2_O_7_ modified electrode displays a clear stepwise increase with successive BPA additions (0.001–425.8 µm) in (0.1 m PB, pH 7.0) at an applied potential of E_pa_ = 0.3 V. A well‐defined increase in current intensity was observed with each successive injection of BPA, confirming fast electron transfer kinetics and stable electrode performance. Further, the calibration plot between the current response and the concentration of BPA is presented in Figure 6b. The modified sensor demonstrates a good linear correlation across the tested range, with regression coefficients of R^2^ = 0.9942 (lower concentrations) and R^2^ = 0.9916 (higher concentrations). The calculated wide linear range (WLR) is 0.001–425.8 µm. These findings affirm that the La_2_Sn_2_O_7_ modified electrode provides an efficient platform for the trace‐level detection of BPA. The following important elements contribute to La_2_Sn_2_O_7_ better analytical performance when compared to other nanomaterials that have been reported: (i) The potent cubic pyrochlore structure is very stable electrochemically and retains its structure even when BPA is sensing repeatedly; (ii) The oxygen‐deficient lattice and interconnected SnO_6_ octahedral network improve the charge‐transfer kinetics at the electrode interface and speed up electron transport; (iii) The surface defects and oxygen vacancies are abundant and serve as effective adsorbents and catalysts, which boost sensitivity and BPA oxidation; and (iv) The presence of Sn^4+^/Sn^2+^ redox couples and Lewis acidic La^3+^ centers together speed up electron transfer, leading to amplified electrochemical signals. Hence, the fabricated electrode can be regarded as a potential candidate for sensitive and reliable electrochemical detection of BPA. Additionally, Table 1 presents comparative electrochemical parameters of recently published reports.

**TABLE 1:** The electrochemical parameters of the La_2_Sn_2_O_7_/SPCE toward BPA detection compared with previously reported electrodes.

Electrodes	Linear range (µM)	LOD (µM)	Technique	Refs.
^a^Cu/CuO‐N‐C	1.0–11.0	0.031	DPV^i^	[40]
^b^Ni‐Cu(PDA)MOF	1–150	0.075	DPV	[41]
CuWO_4_/*f*‐MWCNT	0.05–160	0.04	DPV	[42]
WS_2_‐Fe_3_O_4_‐rGO	0.05–50	0.03	DPV	[43]
^c^SWCNT‐PANI	5–438	4.61	DPV	[44]
^d^GO/MWCNT‐βCD	0.05–5; 5–30	0.006	LSV^j^	[45]
^e^CuNPs@LIGE	0.1–10000	0.033	LSV	[46]
^f^CTAB‐GN‐Cu‐MOF	0.1–800	0.035	DPV	[47]
^g^Thionine‐CB	0.5–50	0.2	AMP^k^	[48]
Fe,Ni‐^h^BTC/CNT	2–50	0.7	DPV	[49]
Cu,Ni‐BTC/CNT	2–50	0.5	DPV	[49]
La_2_Sn_2_O_7_/SPCE	0.001–425.8	0.0014	AMP	This work

^a^ Copper/copper oxide‐decorated N‐doped carbon.

^b^ nickel‐copper pyridine‐2,6‐dicarboxylic acid (PDA) metal organic framework (MOF) anchored carbon nanofiber paper.

^c^ single‐walled carbon nanotubes‐polyaniline.

^d^ graphene oxide and β‐cyclodextrin‐functionalized multi‐walled carbon nanotubes.

^e^ copper nanoparticle decorated laser‐induced graphene‐based electrode.

^f^ cetyltrimethylammonium bromide (CTAB)–graphene (GN)–copper‐organic framework (Cu‐MOF).

^g^ thionine–carbon black.

^h^ trimesic acid‐carbon nanotube (BTC‐CNT).

^i^ differential pulse voltammetry.

^j^ linear sweep voltammetry (LSV).

^k^ amperometric.

To ensure the reliability of the modified sensor, its anti‐interference performance was evaluated in the presence of physiologically relevant species. As shown in Figure 6c, the selectivity of the La_2_Sn_2_O_7_/SPCE sensor toward BPA (20 µm) was investigated in the presence of potential interfering substances at concentrations five times higher than that of BPA. The tested interferents included furaltadone, theobromine, mefenamic acid, carbendazim, metol, dopamine, glucose, Pb^2+^, Hg^2+^, Mg^2+^, Ca^2+^, Cl^−^. All measurements were conducted in 0.1 m PB (pH 7.0) at an applied potential of *E*_pa_ = 0.3 V. These interferents were chosen because their oxidation potentials are close to that of BPA, confirming the high selectivity of the La_2_Sn_2_O_7_/SPCE sensor for BPA detection. The excellent discrimination capability can be attributed to the favorable charge‐transport kinetics and the uniform distribution of surface‐active sites, which selectively enhance BPA oxidation while minimizing interference effects. The chemical structures of the interfering species are shown in Figure S2. Figure 6d demonstrates the reproducibility of four different La_2_Sn_2_O_7_/SPCE electrodes toward BPA (50 µm), showing only a ± 1.94% deviation from the initial response, indicating good reproducibility. The operational stability of the electrode, assessed via amperometric (i‐t) and CV measurements, is presented in Figure 6e,f. The current responses remained stable up to 1000 s and the 100^th^ cycle, showing only ± 8.22% and ± 5.15% variation, respectively, from the initial signal. A study was conducted to investigate the constructed the La_2_Sn_2_O_7_/SPCE sensor's stability over an extended period of time. Over the course of 18 days, the fabricated electrodes were kept at a temperature of 4°C, and the electrochemical signal was monitored at regular intervals. According to Figure S3, the relative signal variation over 18 days was kept under control, falling below ± 7.2%, suggesting that the sensor demonstrated satisfactory long‐term stability.

### 3.7 Real Sample Analysis of La_2_Sn_2_O_7_/SPCE Towards the Spiked BPA Detection

**FIGURE 7:**
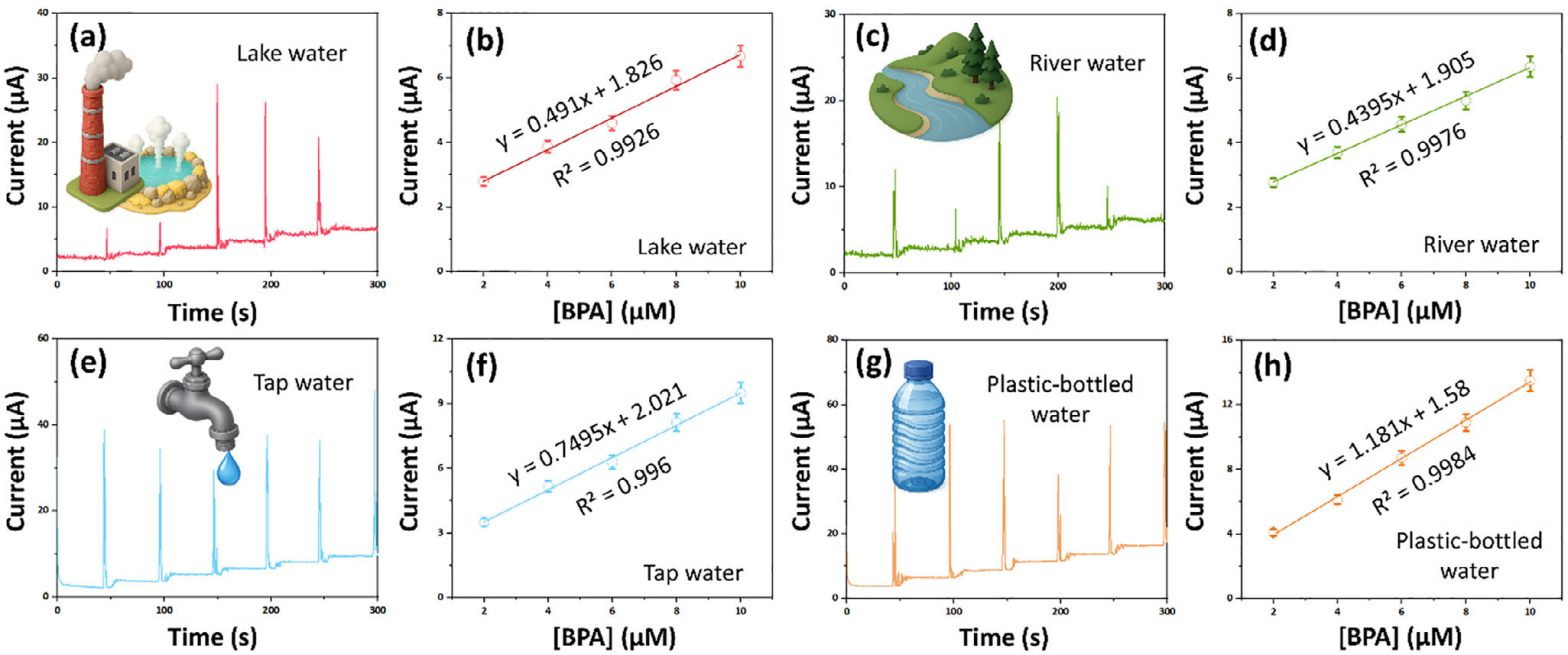
The amperometric current‐time response of the La_2_Sn_2_O_7_/SPCE electrode for (a) lake, (c) river, (e) tap, (g) plastic‐bottle water samples spiked with BPA, and the corresponding anodic peak currents vs. different concentrations of (b) lake, (d) river, (f) tap, (h) plastic‐bottle water samples spiked with BPA.

The practical applicability of the proposed sensor for electrochemical detection of BPA in various water samples‒including lake, river, tap, and plastic‐bottle water‒was evaluated using the amperometric (i‐t) technique. Lake water was collected from Drunken Moon Lake on the National Taiwan University campus (Taipei, Taiwan), while river water was obtained from the Tamsui River near Yanping Riverside Park (Taipei, Taiwan). Tap water samples were collected from the National Taipei University of Technology, and bottled water was purchased from a local convenience store in Taipei, Taiwan. Each water sample was diluted 15‐fold with a pH 7.0 buffer solution and subsequently spiked with a known concentration of BPA. The experimental conditions were consistent with those described in Figure 7a–h and Table S2. The La_2_Sn_2_O_7_/SPCE electrode exhibited excellent recovery rates ranging from ± 91% to 99%, confirming its high accuracy and reliability for BPA detection across different water samples. These recovery values are in good agreement with those obtained using the standard HPLC method (±97%–99.4%), demonstrating the practical applicability of the proposed sensor. Further investigated in our upcoming research when we test the sensor's capabilities with untreated, real‐world water samples from different environments, each with its own unique pH and complex matrix.

### 3.8 Contribution Toward Environmental Protection and BPA Mitigation

The developed La_2_Sn_2_O_7_/SPCE electrode provides an efficient and reliable strategy for addressing the environmental threat posed by BPA. The sensor's high electrocatalytic activity, ultra‐low detection limit, and broad linear range enable accurate quantification of BPA in complex water systems, even at trace levels. Its disposable design, low fabrication cost, and portability make it highly suitable for routine environmental screening and on‐site monitoring. Thus, this work offers a practical platform for real‐time BPA surveillance, contributing to improved pollution assessment and protection of ecological and human health.

## 4 Conclusion

Lanthanum stannate (La_2_Sn_2_O_7_) was successfully synthesized, and detailed analytical and electrocatalytic studies clearly demonstrated the formation of the material, the electronic states of its individual components, improved electron transport, and a higher number of electroactive sites due to the unique interaction between La and Sn. In aquatic samples, the SPCE that had been modified with La_2_Sn_2_O_7_ demonstrated effective electrocatalytic detection and the ability to monitor BPA in real‐time. The electrocatalytic performance of La_2_Sn_2_O_7_/SPCE was modulated systematically, taking into careful consideration pH and interfering species. The capability of the sensor to perform real‐time monitoring was proven by employing a conventional spike‐and‐recovery monitoring technique. In light of the analytical and electrocatalytic data, the following findings were reached:

- La_2_Sn_2_O_7_/SPCE demonstrated high recoveries in various water types as 91%–99% (lake water), ∼97%–99% (river water), ∼92%–99% (tap water), and ∼93%–99% (plastic‐bottled water) samples, with RSD below 3%.
- The modified sensor demonstrated desirable electrocatalytic properties, such as low LOD, wide linear range (WLR), high sensitivity, specificity, reproducible performance, and operational stability for BPA detection.

In conclusion, the La_2_Sn_2_O_7_/SPCE sensor shows great promise as a point‐of‐care (POC) device for the immediate identification of harmful organic contaminants in water samples taken from the environment.

## Conflicts of Interest

The authors declare no conflicts of interest.

## Supporting information




**Supporting File**: gch270088‐sup‐0001‐SuppMat.docx.

## Data Availability

The data that support the findings of this study are available from the corresponding author upon reasonable request.
