# Supplementary material for: Towards Safer Water: A Low‐Cost Disposable Electrochemical Sensor for Bisphenol A Using La2Sn2O7 Nanostructures
Source: Glob Chall. 2026 Jan 14;10(1):e00579. doi: 10.1002/gch2.202500579 (PMC12805055; doi:10.1002/gch2.202500579)
Supplement: Supplementary file 1 — Supporting File: gch270088‐sup‐0001‐SuppMat.docx. [file GCH2-10-e00579-s001.docx]

*Supporting information for*

**Towards Safer Water: A Low-Cost Disposable Electrochemical Sensor for Bisphenol A Using La_2_Sn_2_O_7_ Nanostructures**

Ragu Sasikumar ^a, b, #^, Balasubramanian Akila ^c, #^, Shen-Ming Chen ^c, *^, Jongwon Kim ^d^, Byungki Kim ^a, e, *^

^a^ School of Mechatronics Engineering, Advanced Technology Research Center, Korea University of Technology and Education, Cheonan, Chungnam, 31253, Republic of Korea

^b^ Centre of Molecular Medicine and Diagnostics, Saveetha Dental College and Hospitals, Saveetha Institute of Medical and Technical Sciences, Saveetha University, Chennai 600077, India

^c^ Department of Chemical Engineering and Biotechnology, National Taipei University of Technology, No. 1, Section 3, Chung-Hsiao East Road, Taipei 106, Taiwan

^d^ Department of Mechanical Design Engineering, Korea University of Technology and Education, Cheonan, Chungnam, 31253, Republic of Korea

^e^ Future Convergence Engineering, Korea University of Technology and Education, Cheonan, Chungnam, 31253, Republic of Korea

**Corresponding authors**

Department of Chemical Engineering and Biotechnology, National Taipei University of Technology, No. 1, Section 3, Chung-Hsiao East Road, Taipei 106, Taiwan, ROC. [smchen78@ms15.hinet.net](mailto:smchen78@ms15.hinet.net) (S. -M. Chen)

School of Mechatronics Engineering, Korea University of Technology and Education, Cheonan, Chungnam, 31253, Republic of Korea. [byungki.kim@koreatech.ac.kr](mailto:byungki.kim@koreatech.ac.kr) (B. Kim)

^#^ R. S. and B. A. contributed equally to this work.

*Screen printed carbon electrode and electrochemical sensors*

As production volume increases, the cost per unit of film or screen-printed carbon electrodes decreases. Because of their affordable pricing, these electrodes can be manufactured as disposable or one-time use devices. By disposing of the electrode, it is no longer necessary to clean it after each measurement. A specific electrode application allows the manufacturer to prepare most of it [1, 2]. The disposable screen-printed carbon electrode used in this study costs approximately 45 USD per pack (40 pcs per pkg) at research-scale volumes, and may be cheaper in large-scale production. In contrast, a typical HPLC or GC-MS analysis costs $50 and $100 USD per sample considering instrument time, consumables, and labor [3, 4]. Additionally, the electrochemical workstation costs NT$200,000-300,000 (about 6,000-10,000 USD) [5], comparable to or lower than HPLC/GC-MS instruments [4], and may be reused with multiple disposable sensors. ​

*HPLC Comparison*

The viability of the suggested sensor for the detection of bisphenol A (BPA) in water samples was compared with HPLC analysis. Mobile phase A consisted of DI water with 0.1% formic acid, while mobile phase B consisted of acetonitrile with 0.1% formic acid. Using a C18 column in gradient reversed-phase mode (30% B initial to 90% B), 5-20 μL injection volume, and 0.5 mL/min flow rate. The recoveries in spiked water samples, measured at trace levels (2-10 μg L^-1^) with an RSD <2%, were range of ±98-99.6% according to the HPLC data. These results validate the accuracy of the approach and the sensor's capacity to detect BPA in water in real-time.

**_.
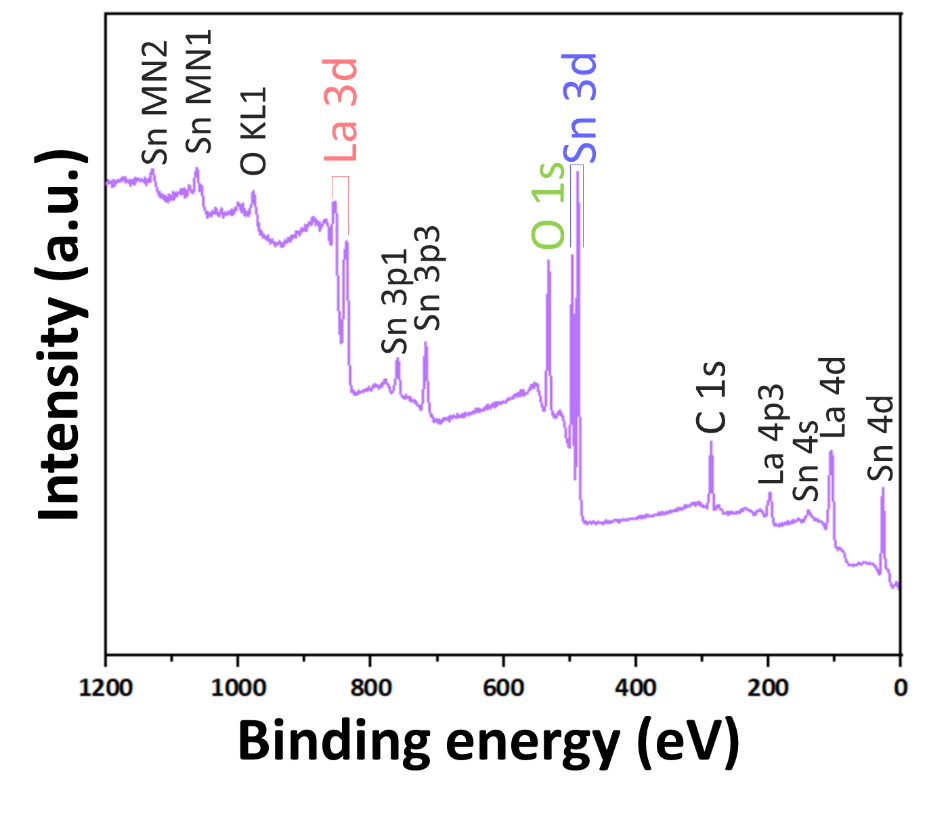
_**

**Figure. S1** XPS survey spectrum of La_2_Sn_2_O_7_ nanoparticles.


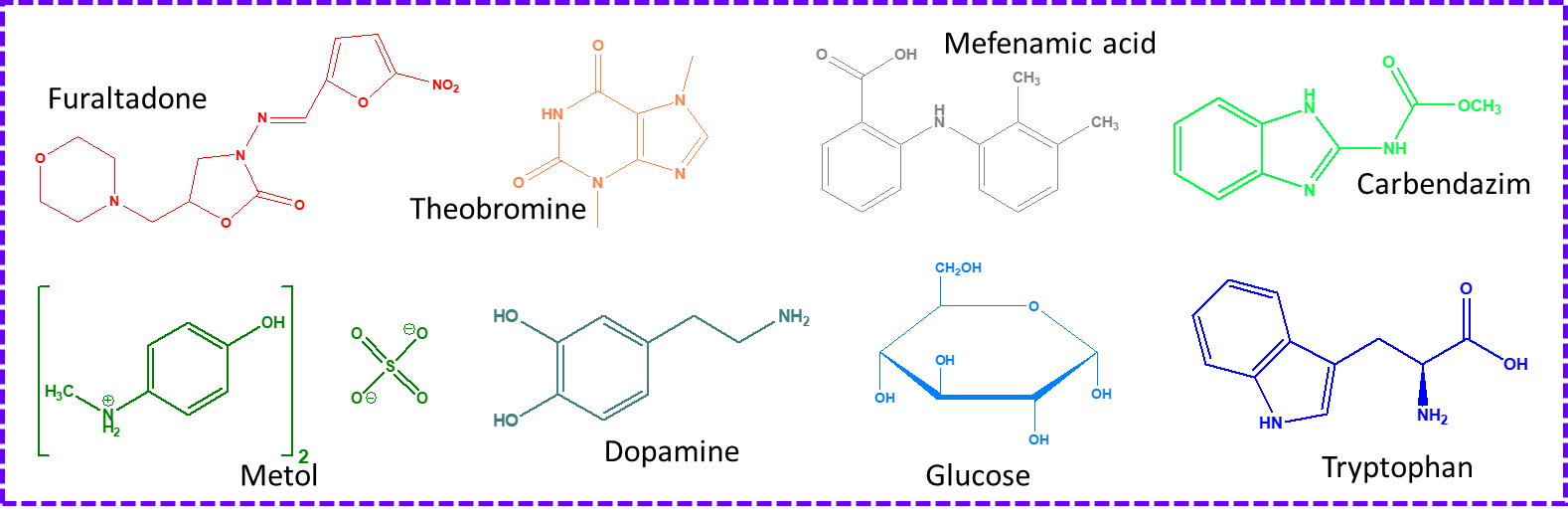


**Figure. S2** Chemical structures of analytes used in selectivity study.

***
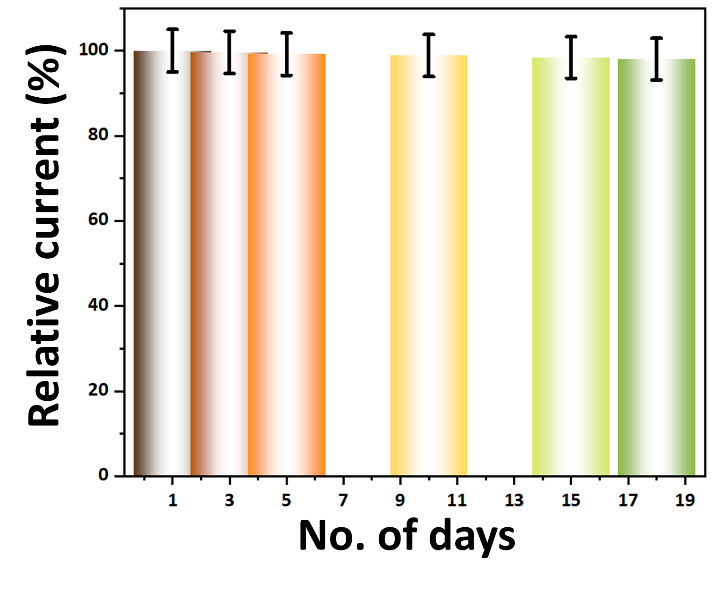
***

**Figure S3.** Long term stability of La_2_Sn_2_O_7_/SPCE in the presence of BPA (Error bars represent the standard deviation (SD) of all measurements).

| **Table S1.** Chemical structure and detailed information of BPA used in this study. | |
| --- | --- |
| Common name | Bisphenol A (BPA) |
| IUPAC name | 4,4’-(Propane-2,2-diyl)diphenol |
| Appearance | White Solid |
| Empirical formula | C_15_H_16_O_2_ |
| Odor | Phenolic |
| Melting and boiling point | 155 ^º^C and 250 – 252 ^º^C |
| Solubility in water (g L^−1^) | 0.3 |
| Solubility in other solvents | Acetone, Methanol, Ethanol, Ethyl Acetate |
| Molecular weight (g mol^−1^) | 228.291 |
| Density (g cm^-3^) | 1.217 |
| Chemical Class | Bisphenols and Diphenylmethanes |
| LD_50_ (median dose) | 4 g kg^-1^ (Mouse) |
| Chemical and Molecular structure | 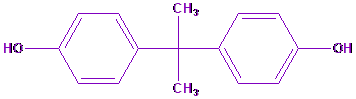  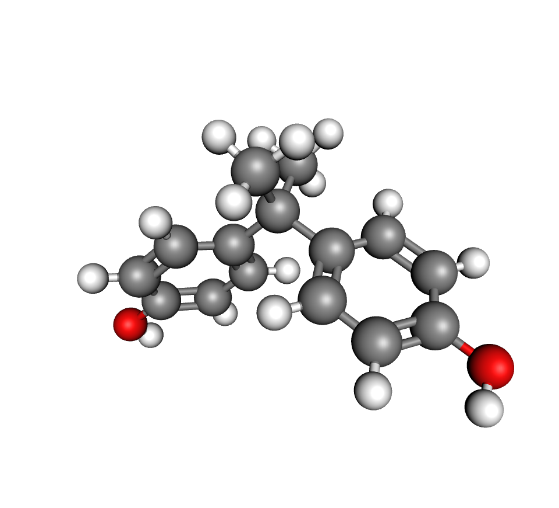 |

**Table S2.** Recovery values of real sample analysis for BPA at La_2_Sn_2_O_7_/SPCE.

| **Samples** | **Added (μM)** | **Found (μM)** | **Recovery (%)** |
| --- | --- | --- | --- |
| **Environmental samples** |  |  |  |
| *Lake water – 1* | - | No detected | - |
| Spiked lake water – 1a | 2 | 1.97 | 98.50 ± 0.74 |
| Spiked lake water – 1b | 4 | 3.91 | 97.75 ± 2.09 |
| Spiked lake water – 1c | 6 | 5.45 | 90.83 ± 2.35 |
| Spiked lake water – 1d | 8 | 7.89 | 98.62 ± 1.27 |
| Spiked lake water – 1e | 10 | 9.54 | 99.10 ± 1.49 |
| *River water – 2* | - | No detected | – |
| Spiked river water – 2a | 2 | 1.93 | 96.5 ± 1.7 |
| Spiked river water – 2b | 4 | 3.85 | 96.25 ± 0.005 |
| Spiked river water – 2c | 6 | 5.98 | 99.66 ± 1.45 |
| Spiked river water – 2d | 8 | 7.78 | 97.25 ± 1.08 |
| Spiked river water – 2e | 10 | 9.96 | 99.60 ± 2.69 |
| *Tap water – 3* | - | No detected | – |
| Spiked tap water – 3a | 2 | 1.99 | 99.50 ± 0.02 |
| Spiked tap water – 3b | 4 | 3.93 | 98.25 ± 1.16 |
| Spiked tap water – 3c | 6 | 5.78 | 96.33 ± 1.09 |
| Spiked tap water – 3d | 8 | 7.95 | 99.37 ± 2.6 |
| Spiked tap water – 3e | 10 | 9.19 | 91.90 ± 0.007 |
| *Plastic-bottled water – 4* | - | No detected | – |
| Spiked plastic-bottled water – 4a | 2 | 1.85 | 92.50 ± 2.45 |
| Spiked plastic-bottled water – 4b | 4 | 3.89 | 97.25 ± 2.31 |
| Spiked plastic-bottled water – 4c | 6 | 5.72 | 95.33 ± 0.18 |
| Spiked plastic-bottled water – 4d | 8 | 7.93 | 99.12 ± 2.06 |
| Spiked plastic-bottled water – 4e | 10 | 9.97 | 99.70 ± 0.12 |

**References**

1. Hayat, A. and Marty, J.L., 2014. Disposable screen printed electrochemical sensors: Tools for environmental monitoring. *Sensors*, *14*(6), pp.10432-10453.
2. Li, M., Li, D.W., Xiu, G. and Long, Y.T., 2017. Applications of screen-printed electrodes in current environmental analysis. *Current Opinion in Electrochemistry*, *3*(1), pp.137-143.
3. Cost of HPLC-MS or GC-MS: <https://resolvemass.ca/cost-of-gc-ms-analysis/>
4. Cost of HPLC-MS or GC-MS: <https://www.mtoz-biolabs.com/what-is-the-cost-of-performing-an-hplc-ms-or-gc-ms-analysis.html>
5. CHI instrument: <https://www.rescienceinc.com/product-page/ch-instruments-chi660c-electrochemical-workstation>
